# Higher levels of physical activity are associated with less evasive coping, better physical function and quality of life in patients with axial spondyloarthritis

**DOI:** 10.1371/journal.pone.0301965

**Published:** 2024-05-17

**Authors:** Marlies Carbo, Davy Paap, Laura van Overbeeke, Freke Wink, Hendrika Bootsma, Suzanne Arends, Anneke Spoorenberg

**Affiliations:** 1 Department of Rheumatology and Clinical Immunology, University of Groningen, University Medical Center Groningen, Groningen, The Netherlands; 2 Department of Physical Therapy, Saxion, University of Applied Sciences, Enschede, The Netherlands; 3 Department of Human Movement Sciences, University of Groningen, University Medical Center Groningen, Groningen, The Netherlands; 4 Department of Rheumatology, Medical Center Leeuwarden, Leeuwarden, The Netherlands; University of Rochester Medical Center, UNITED STATES

## Abstract

**Objective:**

To evaluate daily physical activity (PA) in relation to psychosocial factors, such as anxiety, depression and different types of coping strategies, as well as patient- and disease-related factors in patients with axial spondyloarthritis (axSpA).

**Methods:**

Consecutive outpatients from the Groningen Leeuwarden AxSpA (GLAS) cohort completed the modified Short Questionnaire to assess health-enhancing PA (mSQUASH), Hospital Anxiety and Depression Scale (HADS) and Coping with Rheumatic Stressors (CORS) questionnaires, as well as standardized patient- and disease-related assessments. Univariable and multivariable linear regression analyses and comparison of lowest and highest PA tertiles were performed to explore associations between the HADS, CORS, patient- and disease-related factors and PA.

**Results:**

In total, 84 axSpA patients were included; 60% male, mean age 49 (SD ±14) years, median symptom duration 20 (25^th^-75^th^ percentiles: 12–31) years, mean ASDAS 2.1 (±1.0). Higher PA levels were significantly associated with better scores on patient-reported disease activity (BASDAI), physical function (BASFI) and quality of life (ASQoL). Furthermore, higher levels of PA were associated with less impact of axSpA on wellbeing and lower HADS depression scores. In the multivariable linear regression model, less use of the coping strategy ‘decreasing activities’ (β: -376.4; p 0.003) and lower BMI (β:-235.5; p: 0.030) were independently associated with higher level of PA. Comparison of patients from the lowest and highest PA tertiles showed results similar to those found in the regression analyses.

**Conclusion:**

In this cohort of axSpA patients, higher levels of daily PA were associated with better patient-reported outcomes and lower depression scores. Additionally, the passive coping strategy “decreasing activities” and lifestyle factor BMI were independently associated with PA. Besides anti-inflammatory treatment, coping strategies and lifestyle should be taken into account in the management of individual axSpA patients. Incorporating these aspects into patient education could increase patient awareness and self-efficacy. In the future, longitudinal studies are needed to better understand the complex relationship between patient-, disease- and psychosocial factors associated with daily PA.

## Introduction

Axial spondyloarthritis (axSpA) is a chronic inflammatory rheumatic disease that is characterized by inflammation of the sacroiliac joints and the spine [1]. Symptoms usually start in the second or third decade of life, therefore, there is a long-term burden of disease. The majority of patients with axSpA experience pain, stiffness and fatigue, which can lead to limitations in daily activities, social participation and impaired physical activity (PA) [2–4].

PA includes all movement by skeletal muscles that consume energy and is defined as all activities a person performs. Physical function refers to the ability to perform an activity [5]. Regular PA in patients with axSpA has been shown to decrease pain, improve function and spinal mobility and is protective against fatigue [6, 7]. Therefore, PA is included in the ASAS/EULAR recommendations to be an integral part of standard care for these patients [8]. The level of PA is generally lower in patients with axSpA compared to in healthy controls [9–10]. Specifically patients with axSpA who have high disease activity tend to be less physically active [11, 12]. However, disease activity alone does not fully explain these differences in PA and other aspects, such as psychosocial factors, may also play a role.

Anxiety and depression are more common in patients with axSpA than in the general population. Both are also associated with higher disease activity scores and lower quality of life (QoL) [13–15]. There is evidence that in the general population that being physically active may help prevent depression [16]. Additionally, a study in patients with ankylosing spondylitis (AS) found that depression scores decreased by almost a third after two months of daily home-based exercise [17]. This demonstrates the reciprocal interaction of depression and PA. In the general population, anxiety was also found to be associated with lower PA levels [18]. There is no published research on the relationship between anxiety and PA in patients with axSpA.

In addition to anxiety and depression, the type of coping strategies used by axSpA patients may also influence PA. Coping strategies are ways to manage stressors such as disease or pain. Health-related stressors are generally managed using passive coping strategies (e.g. avoiding the problem) or active coping strategies (e.g. improvement of perception or behavioural adjustment). Evasive coping (a passive coping strategy) is commonly used by patients with chronic back pain, as well as by patients who are suspected of suffering from axSpA [19]. In patients with AS, evasive coping was found to be associated with poor health-related QoL [20]. Data concerning the relation between coping strategies and PA in axSpA is lacking.

Previous research has shown that patients with rheumatoid arthritis (RA) perceived barriers preforming daily PA. Some of these barriers overlap with those observed in the general population, such as a lack of time and the financial costs of exercise. However, other barriers may be more disease related such as pain and fatigue [21, 22]. To our knowledge, there is no data on which patient-, disease- and psychosocial-related factors are associated with lower daily PA in axSpA.

Therefore, the aim of our explorative study was to evaluate the level of PA in relation to psychosocial factors anxiety, depression and different types of coping strategies, as well as patient- and disease-related factors in patients with axSpA.

## Patients and methods

### Patients

All patients from the Groningen Leeuwarden AxSpA (GLAS) cohort visiting the outpatient clinic between November 2018 and May 2019 were asked to participate in this cross-sectional study. The GLAS cohort is a large prospective, longitudinal, observational cohort study in the North of the Netherlands with a standardized assessment protocol according to the recommendations of the Assessment of SpondyloArthritis international Society (ASAS). All participating patients were ≥18 years old and met the modified New York criteria for ankylosing spondylitis (AS) and/or the ASAS classification criteria for axSpA [23]. The GLAS cohort was approved by the local ethics committees of the Medical Centre Leeuwarden (MCL) and the University Medical Centre Groningen (UMCG):TPO 364/604, amendment of PA project was approved at May 17^th^, 2018. All patients provided written informed consent according to the Declaration of Helsinki.

### Assessments

Patient characteristics and disease related assessments were obtained from the standardized Groningen Leeuwarden Axial Spondylarthritis (GLAS cohort during visits at the outpatient clinic. Characteristics and assessments included were: age, gender, HLA-B27 status, symptom duration, AS Disease Activity Score with C-Reactive Protein (ASDAS), Bath AS Disease Activity Index (BASDAI), C-Reactive Protein (CRP), Body Mass Index (BMI), Bath Ankylosing Spondylitis Functional Index (BASFI), Ankylosing Spondylitis Quality of Life (ASQoL) and disease influence on wellbeing in the last week on a numeric rating scale (NRS 0–10). Patients were asked to complete questions about their employment status as well as to complete three additional questionnaires: the modified Short Questionnaire to assess health-enhancing PA (mSQUASH), the Hospital Anxiety and Depression Scale (HADS) and the Coping with Rheumatic Stressors (CORS). All questionnaires have been validated in Dutch.

The mSQUASH is an adaptation of the Dutch version of the SQUASH and is validated in patients with axSpA [24–26]. The mSQUASH measures PA considering an average week in the past month and includes activities related to commuting, work and school, household activities, leisure time and sports. The total amount of minutes per activity is multiplied with a factor for perceived intensity, which is based on metabolic equivalent of task (MET) values of the Ainsworth’s compendium [27]. The mSQUASH score is a product of duration, METs and experienced demand of PA. A higher mSQUASH score indicates a higher level of PA. The mSQUASH score was excluded if the total minutes of activity per day exceeded 960 minutes [24].

The HADS was developed to detect states of anxiety and depression in the outpatient clinic [28]. The HADS consists of seven questions concerning anxiety (HADS-Anxiety) and seven question about depression (HADS-Depression), all questions answered on a Likert scale (0–3). In the literature, a score ≥8 in one of the domains indicates the potential presence of clinically relevant anxiety or depression [29].

The CORS was originally developed and validated for patients with RA and measures coping strategies for three stressors related to chronic rheumatic diseases: pain, limitations and dependency [30]. The questionnaire consists of 61 questions on a Likert scale (1–4). The coping strategies for pain (25 questions) in the CORS are ‘comforting cognitions’, ‘decreasing activities’ and ‘diverting attention’. The coping strategies used for limitations (23 questions) are ‘pacing’, ‘optimism’ and ‘creative solution seeking’ and for dependency (13 questions) ‘accepting dependence’ and ‘showing consideration’. The total score is computed by calculating the sum score of questions belonging to a specific coping strategy.

### Statistics

Statistical analysis was performed with IBM SPSS Statistics 23.0 (SPSS, Chicago, IL, USA). Results were expressed as number of patients (%), mean ± SD and median (25^th^-75^th^ percentiles) for categorical, normally distributed and non-normally distributed data, respectively. Univariable linear regression analysis was used to analyse the association of PA (continuous dependent variable) with patient characteristics (gender and BMI), clinical outcome measures (BASDAI, fatigue, ASDAS, BASFI, disease influence of wellbeing and ASQoL), anxiety, depression and coping strategies. Variables with p-value < 0.1 in the invariable linear regression analysis were tested in the multivariable linear regression model. ASDAS was used in the multivariable model instead of BASDAI, since ASDAS includes both objective (CRP) and patient-reported (BASDAI questions and patient global) assessments of disease activity [31]. Multivariable linear regression analysis with forward inclusion was performed to investigate independent associations with PA. Multicollinearity was tested before entering the variables into the multivariable linear regression analysis.

There are no validated cut-off points for activity categories in the mSQUASH. Therefore, total mSQUASH scores were categorically divided into tertiles to compare patients with the lowest, intermediate and highest PA level. The Mann-Whitney U test or Pearson’s Chi-Square test was used to identify significant differences between these groups. P-values 0.05 were considered statistically significant.

## Results

In total, 84 out of 85 patients were eligible for analyses. One patient was excluded from the study because PA exceeded the 960 minutes per day according to the mSQUASH protocol. The mean age was 49 (±14) years, 60% were male; median symptom duration was 20 years (12–31), and 71% were HLA-B27 positive. Patients had a mean ASDAS score of 2.1 (±1.0), mean BASDAI score of 4.0 (±2.5) and median BMI of 26.9 (23.9–29.8). All patient characteristics are shown in Table 1. Patients reported to perform a median 750 (420–1380) minutes of light intensive PA, 1440 (366–2400) minutes of moderate- intensive PA and 120 (0–420) minutes of vigorous- intensive PA per week. The median PA mSQUASH score was 8496 (7440–9510). More than half of the patients (57%) participated in sports, mostly gym exercise.

**Table 1:** Patient characteristics.

		All patients (n = 84)
Age (years)		49 ± 14
Gender (male), n (%)		50 (60%)
HLA-B27+		60 (75%)
Symptom duration (years)		20 (12–31)
ASDAS		2.1 ± 1.0
BASDAI (0–10)		4.0 ± 2.5
CRP (mg/L)		1.9 (1.0–4.4)
BMI (kg/m^2^)		26.9 (23.9–29.8)
BASFI (0–10)		3.0 (1.1–5.7)
ASQoL (0–18)		4.2 (2.0–10.0)
Disease influence on wellbeing (0–10)		4.0 (1.0–6.0)
Paid employment		49 (59%)
mSQUASH total score		8543 (5213–11075)
HADS	Anxiety (0–21)	5 (3–7)
	Depression (0–21)	3 (2–6)
CORS	Coping with pain	
	• Comforting cognitions (9–36)	25 (22–28)
	• Decreasing activities (8–32)	18 (15–21)
	• Diverting Attention (8–32)	19 (15–21)
	Coping with limitations	
	• Optimism (5–20)	15 (13–17)
	• Pacing (10–40)	24 (21–29)
	• Creative solution seeking (8–32)	20 (17–23)
	Coping with dependence	
	• Accepting one’s dependence (6–24)	13 (10–16)
	• Showing consideration (7–28)	16 (14–18)

Data presented as number of patients (%), mean ± SD or median (25^th^-75^th^ percentiles).; HLA-B27+: human leukocyte antigen B27-positive; BASFI: Bath Ankylosing Spondylitis Functional Index; ASQoL: Ankylosing Spondylitis Quality of Life; HADS: Hospital Anxiety and Depression Scale; CORS: Coping with Rheumatic Stressors.

Median HADS-Anxiety score was 5 (3–7) and median HADS-Depression score was 3 (2–6). The HADS indicated the potential presence (subscore ≥8) of anxiety in 19 (22%) patients and of depression in 13 (15%) patients. From the eight coping strategies in the CORS questionnaire, ‘comforting cognitions’ and ‘pacing’ were used most often with median scores of 25 (22–28) and 24 (21–29), respectively (Table 1).

### Patient-, disease- and psychosocial-related factors associated with PA

Univariable analysis showed that lower patient-reported disease activity (BASDAI), better physical function (BASFI) and better disease-related QoL (ASQoL) were significantly associated with higher levels of PA. Less impact of axSpA on general wellbeing and a lower score on the HADS-Depression scale were also significantly associated with higher levels of PA. On the other hand, the coping strategies ‘decreasing activities’ and ‘pacing’ were more frequently used by patients who were less physically active (Table 2).

**Table 2:** Univariable and multivariable regression analyses exploring the relation of patient-, disease- and psychosocial- variables and total PA.

	Univariable			Multivariable	
	β (95% CI)	R^2^	p	β(95% CI)	p
Gender (male)	-1557 (-3678;563)	0.16	0.148		^a^
ASDAS	-801 (-1876;272)	0.17	0.141		^b^
BASDAI (0–10)	-460 (-902;-19)	0.23	0.041		^a^
CRP	-.0.33 (-173;128)	<0.01	0.771		^a^
BMI	-146 (-355;62)	0.19	0.116	-236 (-451; -20)	0.030
BASFI	-644 (-1087;-202)	0.33	0.005		^c^
Disease influence on wellbeing (0–10)	-456 (-867; -45)	0.26	0.030		^a^
HADS					^a^
• Anxiety (0–21)	-376 (-663; -89)	0.28	0.203		
• Depression (0–21)	-176 (-450; 97)	0.14	0.011		^a^
CORS					
• Comforting cognitions (9–36)	-15 (-228;198)	0.02	0.888		^a^
• Decreasing activities (8–32)	-427 (-644;-208)	0.40	-0.0010.836	-376 (-622; -131)	0.003
• Diverting Attention (8–32)	-25(-269;271)	0.02			^a^
• Optimism (5–20)	-37 (-302;378)	0.03	0.825		^a^
• Pacing (10–40)	-251 (-411;-90)	0.34	0.0031		^c^
• Creative solutions (8–32)	-95 (-313;123)	0.10	0.388		^a^
• Accepting dependence (6–24)	-2793 (-588;2)	0.22	0.051		^a^
• Showing consideration (7–28)	-275(-601;50)	0.19	0.097		^c^
ASQoL (0–18)	-331 (-571;-91)	0.32	0.0071		^a^

CI: confidence interval;; ASDAS: Ankylosing Spondylitis Disease Activity Score; BASDAI: Bath Ankylosing Spondylitis Disease Activity Index CRP: C-reactive protein; BMI: Body Mass Index;BASFI: Bath Ankylosing Spondylitis Functional Index;: Hospital Anxiety and Depression Scale; CORS: Coping with Rheumatic Stressors;ASQoL: Ankylosing Spondylitis Quality of Life.

^a^ Variable was not selected due to a P-value ≥ 0.1 in the univariable analysis.

b BASDAI was not entered into the multivariable model due to being an overlapping construct with ASDAS.

c Variable was not entered into the multivariable model due to multicollinearity.

^b^ ASDAS was entered in the multivariate model instead of BASDAI, as explained in the method section

^c^ Not entered into the multivariate model due to multicollinearity.

Although ASDAS was not significantly associated with PA, ASDAS was entered in the multivariable model instead of BASDAI, as explained in the method section. BASFI and ‘pacing’ were not entered in the multivariable model due to multicollinearity (with BASDAI and decreasing activities, respectively). In the multivariable linear regression model, the coping strategy ‘decreasing activities’ (β: -376.4; 95%CI: -621.9; -130.8, p-value: 0.003) and BMI (β:-235.5; 95%CI: -450.9; -20.0, p-value: 0.030) were independently associated with the level of PA.

### Patient-, disease- and psychosocial-related factors stratified for the tertiles of PA

The mSQUASH PA levels reported by the axSpA patients were stratified into tertiles; low, intermediate and high (Table 3). Patients in the highest PA tertile had significantly lower disease activity scores (BASDAI and ASDAS), lower BMI, better physical function (BASFI), better disease-related QoL (ASQoL) and less perceived impact effect of axSpA on wellbeing compared to patients in the lowest PA tertile. Additionally, the patients in the highest PA tertile were significantly more likely to be employed, reported significantly lower HADS-Anxiety and Depression scores and used significantly less often the coping strategies ‘decreasing activities’ and ‘pacing’ than patients in the lowest PA tertile. Similar results or a trend toward these results were found when comparing the low and intermediate PA tertile and the intermediate and high PA tertile (Table 3).

**Table 3:** Comparison of patient-, disease- and psychosocial- variables stratified for tertiles of PA.

		mSQUASH-tertiles		
		**Low** n = 27 PA range 1030–6075	**Intermediate** n = 27 PA range 6210–10370	**High** n = 28 PA range 10725–21585
Age (years)		48.5 ± 14.6	50.8 ± 14.0	46.4 ± 12.8
Gender (male), n (%)		14 (52.0)	15 (55.6)	21 (75.0)* ^‡^
HLA-B27+, n (%)		16 (70.0)	20 (77.0)	24 (86.0)
Symptom duration (years)		17.5 (11.5–27.5)	20.0 (13.0–35.0)	19.5 (11.8–29.0)
ASDAS		2.6 (1.9–3.1)	1.9 (1.0–2.8) †	2.1 (1.1–2.7)*
BASDAI (0–10)		5.1 (3.4–6.8)	3.0 (1.1–4.9) †	2.2 (1.4–5.2)*
CRP (mg/L)		1.8 (0.8–3.2)	2.8 (1.1–10.0)	1.2 (0.7–4.3)
BMI (kg/m2)		27.9 (26.3–30.8)	26.1 (23.7–30.3) †	25.7 (22.7–27.8)*
BASFI (0–10)		4.8 (2.6–7.0)	2.1 (0.7–4.7) †	2.7 (1.0–4.0)*
ASQoL (0–18)		9.3 (3.3–13.0)	3.6 (0.0–8.1) †	4.0 (1.0–6.9)*
Disease influence on wellbeing (0–10)		6.0 (4.0–8.0)	3.0 (1.0–5.0)†	3.0 (1.0–6.0)*
Paid employment, n (%)		9 (35.0)	15 (55.6)	24 (86.0)*^‡^
HADS	Anxiety	6 (4–10)	4 (2–6)†	4 (2–4)*
	Depression	5 (3–9)	2 (1–4)†	3 (2–3)*
CORS	Coping with pain			
	• Comforting cognitions (9–36)	26 (23–28)	26 (21–28)	25 (23–30)
	• Decreasing activities (8–32)	21 (18–23)	17 (13–20)†	16 (14–18)*
	• Diverting Attention (8–32)	19 (15–21)	19 (14–21)	19 (16; 20)
	Coping with limitations			
	• Optimism (5–20)	15 (13–16)	15 (14–17)	15 (13–17)
	• Pacing (10–40)	27 (24–30)	22 (20–28)	22 (17–26)*
	• Creative solution seeking (8–32)	21 (18–23)	20 (15–22)	19 (17–24)
	Coping with dependence			
	• Accepting dependence (6–24)	14 (12–16)	11 (9–15)	12 (10–16)
	• Showing consideration (7–28)	16 (15–18)	16 (13–17)	17 (14–18)

Patients are divided into three levels of PA: low PA, intermediate PA and high PA. Data presented as number of patients (%), mean ± SD or median (25th-75th percentiles). HLA-B27+, human leukocyte antigen B27-positive; BASDAI, Bath Ankylosing Spondylitis Disease Activity Index; ASDAS, Ankylosing Spondylitis Disease Activity Score; CRP, C-reactive protein; BASFI, Bath Ankylosing Spondylitis Functional Index; ASQoL, Ankylosing Spondylitis Quality of Life. HADS, Hospital Anxiety and Depression Scale; CORS, the Coping with Rheumatic Stressors.

* P-values ≤ 0.05 for highest PA group compared to lowest PA group.

† P-values ≤ 0.05 for intermediate PA group compared to lowest PA group.

‡ P-values ≤ 0.05 for highest PA group compared the intermediate PA group.

## Discussion

This study was conducted to gain further insight into the relation of patient-, disease- and psychosocial-related factors with daily PA in patients with axSpA. Patients preformed 95% of their reported activity in moderate to light intensity. More than half of the axSpA patients reported participating in sports, most often gym exercises. Higher levels of PA were associated with better scores on patient-reported outcomes including disease activity (BASDAI), physical function BASFI) and QoL (ASQol). The associations of PA with disease activity and physical function were also observed in previous research, as described in a recent update of the ASAS/EULAR recommendations for axSpA [7]. Consistent with our finding, a Norwegian cross-sectional study in patients with axSpA showed that lower self-reported PA was associated with lower health related QoL [32]. In our study, we also found that lower BMI was independently associated with a higher level of PA. These results are in line with a cross-sectional study, which showed that a lower BMI was associated with a higher level of PA assessed with an accelerometer in both AS patients and controls [33]. Concerning psycho-social factors in relation to PA, we found that lower scores on the HADS-depression subscale and less frequent use of the coping strategies ‘decreasing activities’ and ‘pacing’ were associated with higher reported level of daily PA. This association is well documented in the general population and in patients with RA, but has not been previously reported in axSpA [34, 35]. In our study, median HADS scores were comparable to those of the general Dutch population [36]. The reported anxiety and depression scores in other international axSpA populations were generally higher, but comparisons are difficult due to potential cross-cultural differences in perception and social beliefs [14, 17]. Additionally, a cross-sectional Dutch multicentre study in axSpA patients showed that male gender, unemployment status, lower income and higher BASDAI were associated with depression in AS. The extent to which patients experience control over their life and disease appears to play an important role in the experience of depressive symptoms, as measured by the HADS [37]. In our study, we also found that patients in the lowest PA tertile were less likely to have paid employment.

As far as we know, this is the first study exploring the relation of coping strategies and PA in patients with axSpA. We found that the passive coping strategies ‘decreasing activities’ and ‘pacing’ were less likely applied by axSpA patients with higher PA levels. The coping strategy ‘decreasing activities’ was independently associated with the level of PA. Previous studies in patients with AS did report and association of the coping strategies ‘decreasing activities’ and ‘pacing’ with physical function [38, 39]. The operant learning theory provides a theoretical framework to explain the increased use of these coping strategies assuming that behaviour is subject to environmental influences and that coping strategies can cause dysfunctional behaviour. For example, pain experienced during PA can lead to lower physical efforts to avoid this pain [40]. This lower effort may lead to even worse experienced symptoms and more (fear of) pain, resulting in a vicious circle of avoiding PA [41, 42]. This is supported by the finding that up to 30% of patients with axSpA believe that PA triggers a disease flare [40, 43]. On the other hand, a recent randomized controlled trial in axSpA patients showed that high intensity PA reduced disease symptoms (e.g. pain, stiffness, fatigue and inflammation) and was not related to disease activity flares [44].

The benefits of regular PA on a wide range of organs and systems are well-documented. In the context of inflammatory rheumatic diseases, the immune-modulatory effects of exercise are likely contributors to reduced disease activity [45]. The effects of regular PA on the immune system are multifaceted, and are generally considered to be anti-inflammatory [46–48]. The mechanism how exercise elicit these anti-inflammatory effects are not yet completely clear. Amongst others, physical exercise may lead to upregulation of mRNA encoding for IL-6 by skeletal muscle cells, followed by its subsequent release. In the context of exercise, IL-6 acts as an anti-inflammatory myokine, resulting in the production of IL-1 receptor antagonist (IL-1RA) and IL-10, two cytokines capable of inhibiting inflammatory processes [48]. Prolonged exercise may also result in reduced Toll-like receptor (TLR) expression and signalling by monocytes, particularly TLR2 and TLR4, leading to decreased pro-inflammatory cytokine production [47]. Furthermore, a reduction in the total amount of adipose tissue by regular PA may reduce low-grade systemic inflammation caused by pro-inflammatory cytokines produced by adipose tissue. Collectively, these effects may dampen systemic inflammation, resulting in reduced disease activity and better outcomes.

A limitation of our study is the explorative cross-sectional design, in which it is not possible to unravel the longitudinal and causal interrelationships of patient-, disease and psychosocial-factors with PA. Although our study has a limited sample size, we found consistent results in all analyses (based on our regression models and compering tertiles). To confirm our findings and rule out the possibility of a Type I error due to multiple testing, replication in future studies is warranted. It should be kept in mind that the HADS is a screening instrument and clinical confirmation of an anxiety or depression diagnosis is required by a medical professional [49]. In general, our findings demonstrate a preferable overall outcome in patients with axSpA with a higher level of daily PA. Recently, a questionnaire to assess facilitators and barriers to perform PA for patients with axSpA has been developed and validated (Inflammatory arthritis Facilitators And Barriers to PA questionnaire), which could be helpful in the management of axSpA patients in daily clinical practise as well as for the development of patient- centred interventions directed to increase levels of PA [50]. Disease perceptions, which may influence coping and PA, were not included in our study [20]. In general, using PA questionnaires introduces the risk of a recall bias and especially the risk of overestimation of the intensity of PA [24, 42]. However, the mSQUASH has shown to be a valid, reliable and responsive questionnaire for measuring PA in axSpA [51, 52].

In summary, in our cross-sectional daily life axSpA cohort study, higher levels of daily PA were associated with better patient-reported outcomes and better psychosocial status. Additionally, the passive coping strategy ¨decreasing activities¨ and lifestyle factor BMI were independently associated with PA. Besides anti-inflammatory treatment, coping strategies and lifestyle should be taken into account in the management of individual axSpA patients. Incorporating these aspects into patient education could enhance patient awareness and self-efficacy.

In the future, longitudinal studies are needed to better understand the complex relationship between patient-, disease- and psychosocial factors associated with daily PA.
